# Deep versus moderate hypothermia in pediatric cardiac surgery: Systematic review and meta-analysis of early postoperative outcomes

**DOI:** 10.1177/02676591261477954

**Published:** 2026-08-16

**Authors:** Ioannis Zoupas, Jared M. Covell, Julieta Cruz Naranjo, Blaz Podgorsek, Peter C. Chen, Damien J. LaPar, Christopher E. Greenleaf

**Affiliations:** 1 Division of Pediatric and Congenital Cardiac Surgery, Department of Cardiothoracic and Vascular Surgery, Children’s Memorial Hermann Hospital, 12340 The University of Texas Health Science Center at Houston, Houston, TX, USA; 2 Department of Cardiothoracic and Vascular Surgery, Memorial Hermann Hospital, 12340 The University of Texas Health Science Center at Houston, Houston, TX, USA

**Keywords:** hypothermia, DHCA, MH, cerebral perfusion, pediatric, congenital

## Abstract

**Introduction:**

Deep hypothermia (DH) has been widely adopted for cerebral and visceral protection during pediatric cardiac surgery, but concerns about perioperative complications remain. This meta-analysis aims to evaluate the impact of DH compared to moderate hypothermia (MH) on outcomes in pediatric cardiac surgery patients.

**Methods:**

Following a PRISMA-compliant systematic search in PubMed, Cochrane, and Embase, eligible studies comparing DH (<22°C) with MH (22–30°C) in pediatric cardiac surgery were assessed. Risk of bias was evaluated using the ROBINS-I tool.

**Results:**

Five comparative studies with 448 patients (250 in the MH group and 198 in the DH group) were included. Baseline characteristics were similar between groups, with the majority of patients undergoing aortic arch repairs. Perioperative variables were also similar, except for cross-clamp time, which was significantly longer in the DH group (*p* = 0.02), and circulatory arrest was more frequently used in DH patients (*p* = 0.01). No significant differences were found in overall postoperative complication rates, acute kidney injury, 24-h chest drainage volume, or in-hospital mortality. However, the incidence of early postoperative neurological complications was significantly higher in the DH group (*p* < 0.001).

**Conclusions:**

Moderate hypothermia may be associated with a significantly lower incidence of postoperative neurological complications compared to deep hypothermia, while overall in-hospital mortality and morbidity rates remained similar between the two groups. Taken together, these findings support moderate hypothermia with regional perfusion as a clinically feasible and well-tolerated alternative to deep hypothermia in pediatric cardiac surgery.

## Introduction

Deep hypothermia (DH) has been employed for decades in pediatric cardiac surgery as a means of protecting the brain and visceral organs during complex interventions, including aortic arch reconstruction and the arterial switch operation.^1,2,3^ DH has demonstrated efficacy by reducing metabolic rate and oxygen consumption, while facilitating a clear, bloodless operative field that supports precise surgical repair.^4,5^

However, despite historically favorable outcomes, DH has well-documented associations with postoperative complications such as neurological injury, developmental delay, coagulopathy, respiratory and renal dysfunction, and an exaggerated systemic inflammatory response.^3,6^ Deep hypothermia with longer circulatory arrest durations are associated with higher rates of EEG seizures and neurodevelopmental delay, reinforcing the importance of minimizing DH circulatory arrest time.^1^ Consequently, North American centers have shifted away from DH and circulatory arrest, favoring moderate hypothermia (MH) often combined with regional perfusion strategies such as selective antegrade cerebral perfusion (SACP).^3,7^ Moderate hypothermia combined with cerebral perfusion may reduce the incidence of neurological injury, including long-term neurodevelopmental impairment.^8,9^

Despite changes in practice patterns, clear benefits of conventional DH vs MH have yet to be established. Drawing inspiration from other studies, including a North American registry study,^10^ we conducted this meta-analysis comparing postoperative outcomes between deep versus moderate hypothermia aiming to address the need for more granular data from around the globe.

## Materials and methods

### Study design and selection process

The present systematic review and meta-analysis was conducted in accordance with the PRISMA (Preferred Reporting Items for Systematic Reviews and Meta-analyses) recommendations^11^ and was prospectively registered in the PROSPERO database (registration number: CRD420251024016). We used the PICO (Population/Participants, Intervention, Comparison and Outcome) criteria to shape our research question:

1. **Population/Participants:** Pediatric patients undergoing cardiac surgery with the use of hypothermia (<30°C)
2. **Intervention:** Pediatric patients undergoing cardiac surgery with deep hypothermia (DH) (<22°C)
3. **Comparison:** Pediatric patients undergoing cardiac surgery with moderate hypothermia (22–30°C)
4. **Outcomes:** The primary outcome was early (<30 days) survival. The secondary outcomes were perioperative and post-operative complication rates.

### Study selection criteria

Eligible studies included randomized controlled trials, non-randomized prospective clinical studies, retrospective clinical studies, and cohort studies with more than 10 patients. Only articles published in English and reporting clinical characteristics and outcomes of pediatric patients undergoing cardiac surgery with either deep hypothermia (DH) or moderate hypothermia (MH) were considered. We included comparative, two-arm studies directly comparing these two hypothermic strategies.

Studies were excluded if they: (i) were published in a language other than English; (ii) were irrelevant to the research question; (iii) consisted of reviews, meta-analyses, systematic reviews, editorials, letters to the editor, comments, personal opinions, or errata; and (iv) reported outcomes for only one of the techniques without a comparator. For instances where multiple manuscripts analyzed overlapping patient populations, only the study with the largest cohort or the longest follow-up was retained.

### Search strategy

A systematic search of MEDLINE/PubMed, Embase, and the Cochrane Library was conducted (last search: January 14, 2026). The search algorithm was: (deep OR DHCA) AND (moderate OR MHCA) AND (“hypothermic circulatory arrest” OR HCA OR hypotherm*) AND (pediatric OR infant* OR neonat* OR congenital), with no restrictions on publication date. Title and abstract screening, as well as full-text eligibility assessment, were independently performed by two investigators (IZ, JCN). Disagreements were resolved through discussion with a third reviewer (CEG). Additional eligible studies were identified through manual reference screening (snowball method)^12,13^. Rayyan software^13^ was used for reference management and study selection.

### Data extraction and risk of bias assessment

Data were independently extracted by three investigators (IZ, JC, JCN) into a predesigned table, including baseline and perioperative characteristics. Risk of bias in non-randomized comparative studies was evaluated using the Risk of Bias in Non-Randomized Studies of Interventions (ROBINS-I) tool.^14^ Follow-up adequacy was defined as at least 12 months postoperatively. Studies were categorized as having low, moderate, serious, or critical risk of bias. Disagreements were resolved by consultation with a fourth investigator (CEG). Each study was assessed across seven ROBINS-I domains. The overall risk of bias was determined by the most severe domain-level judgment, in accordance with the ROBINS-I guidance. Studies were categorized as low risk of bias when all domains were judged as low; moderate when at least one domain was moderate but none serious or critical; serious when at least one domain was serious; and critical when at least one domain was critical.

### Statistical analysis

For continuous variables, means and standard deviations were extracted or calculated; categorical variables were summarized as frequencies and percentages. When studies reported medians with ranges, means and standard deviations were estimated using the method of Hozo et al.^15,16^; when medians with interquartile ranges were reported, the method of Wan et al.^16^ was applied. Extracted data were organized into summary tables, and pooled effect estimates were calculated. All statistical analyses were performed using Stata/SE 19.0 (StataCorp, College Station, TX, USA).

To compare baseline features and outcomes between the two techniques, we used Standardized Mean Difference (SMD) for continuous^17^ and Odds Ratio (OR) for categorical variables reported in the included studies. P-values smaller than 0.05 were considered statistically significant.

Pooled baseline characteristics are presented as descriptive summaries and should be interpreted with caution, as between-study heterogeneity in patient populations, surgical indications, and institutional practices may limit the generalizability of aggregate estimates. Specifically, the definition of postoperative neurological events varied across studies, and a detailed breakdown of study-specific neurological outcome definitions is provided in Supplemental Table 1. More specifically, across the included studies, only two provided granular data regarding the specific neurological event, but all of them pooled the data into cumulative terms, including intracranial bleeding, seizures, delayed recovery of consciousness, coma, sensory loss, neuroimaging changes, intracerebral edema, thrombosis, and spinal cord injury.

## Results

### Study and patient characteristics

The database search yielded 338 potentially eligible articles prior to removal of duplicates (n = 118). Following Title and Abstract screening, 14 studies were extracted for full-text evaluation. No additional articles were identified using citation searching. After full-text assessment and patient overlap checking, five studies met our eligibility criteria, as summarized in the PRISMA flowchart (Supplemental Figure 1). PRISMA checklists for both the abstract and the full text are provided in Supplemental Tables 2 & 3 All manuscripts were comparative cohort studies.^3,7,18,19,20^ One manuscript was excluded due to patient overlap.^21^

A total of 448 patients from five comparative cohort studies met inclusion criteria, representing a limited evidence base that constrains statistical power, particularly for less common outcomes such as mortality. This small number of included studies and patients, combined with heterogeneity in surgical era, lesion type, and perfusion technique, limits the precision of pooled estimates and the strength of conclusions that can be drawn.

Temperature was measured rectally in the entirety of the patient cohort. Among them, 198 patients received DH and 250 underwent surgery with MH. All MH patients were supported with cerebral perfusion, either in the form of selective antegrade cerebral perfusion (SACP) or full-flow CPB. About 25% of DH patients were supported with SACP, about 70% underwent circulatory arrest and the rest were supported with full flow CPB. Individual characteristics of each study are exhibited in Table 1. Pooled demographic and medical history features are provided in Table 2, while peri-operative details and post-operative complications are described in Table 3. Finally, Standardized Mean Differences (SMDs) for continuous variables, and categorical values with Odds Ratios (OR) for DH vs. MH are shown in Table 4. SMDs and ORs were calculated when a variable was available in at least three of the included studies, and forest plots are provided in Figure 1 for Odds Ratios and Figure 2 for SMDs. P-values, when available, were reported for these tests along with summative statistical comparisons for DH vs. MH.

**Table 1.** Study characteristics of the included manuscripts.

Author	Year	Journal	Center	Country/Region	Design	Study period	n (%)	MH (%)	DH (%)
Abdul-Khaliq et al.	2002	Perfusion	German heart institute Berlin, Berlin	Germany	RSC	NR	47 (100)	29 (61.7)	18 (38.3)
Rastan et al.	2008	EJCTS	University of Leipzig, Leipzig	Germany	RSC	1998-2006	100 (100)	51 (51)	49 (49)
Kornilov et al.	2015	EJCTS	Novosibirsk state Research institute of circulation pathology, novosibirsk	Russia	RSC	2004-2014	62 (100)	35 (56.5)	27 (43.5)
Xie et al.	2020	Scientific reports	Chongqing medical university, chongqing	China	RSC	2008-2017	114 (100)	61 (53.5)	53 (46.5)
Bottcher et al.	2021	WJPCHS	German heart center Berlin, Berlin	Germany	RSC	2007-2019	125 (100)	74 (59.2)	51 (40.1)

Abbreviations: RSC = Retrospective cohort, NR = Not Reported

**Table 2.** Demographics and preoperative diagnosis.

Author	Ν	Age (SD) (days)	Weight (SD) (Kg)	Preoperative diagnosis			
	MH/DH	MH/DH	MH/DH	Arch pathologies (N, %)	VSD (N, %)	TGA (N, %)	DORV (N, %)
				MH/DH	MH/DH	MH/DH	MH/DH
Abdul-Khaliq et al.	29/18	330 (1650)/147 (240)	6.2 (3.3)/4.8 (2.4)	0 (0)/1 (5.6)	6 (20.7)/3 (16.7)	3 (10.3)/14 (77.8)	2 (6.9)/0 (0)
Rastan et al.	51/49	NR/NR	NR/NR	2 (3.9)/6 (12.2)	14 (27.5)/19 (38.8)	51 (100)/49 (100)	NR/NR
Kornilov et al.	35/27	20.8 (25.7)/38.5 (64.5)	3.4 (0.8)/4.1 (3.2)	35 (100)/27 (100)	16 (45.7)/9 (33.3)	9 (25.7)/4 (14.8)	0 (0)/1 (3.7)
Xie et al.	61/53	136 (92.3)/117 (83.3)	5.3 (2.2)/5.3 (2.2)	61 (100)/53 (100)	41 (67.2)/35 (66.0)	0 (0)/0 (0)	4 (6.6)/6 (11.3)
Bottcher et al.	74/51	7.7 (4.5)/5.7 (2.3)	3.4 (0.5)/3.2 (0.7)	74 (100)/51 (100)	NR/NR	NR/NR	NR/NR
Percentage (categorical values) or mean ± SD (continuous variables)	250/198	96.3 (627.4)/68.3 (99.9)	4.4 (1.8)/4.3 (2.1)	172 (77.8)/138 (76.7)	77 (34.8)/66 (36.7)	63 (28.5)/67 (37.2)	6 (2.7)/7 (3.9)

Abbreviations: NR = Not Reported, SD = Standard Deviation, MH = Moderate hypothermia, DH = Deep hypothermia, VSD = Ventricular septal defect, TGA = Transposition of the great arteries, DORV = Double outlet right ventricle.

**Table 3.** Perioperative details and early postoperative outcomes.

Author	Total CPB time (SD) (minutes)	Cross - clamp time (SD) (minutes)	Minimum rectal temper-ature (SD) (celsius)	Use of SACP (n, %)	Use of FFCPB (n, %)	Use of CA (n, %)	ICU LOS (SD) (days)	Ventil-ation time (SD) (hours)	24h chest drain-age (SD) (ml)	Complic-ations (overall) (n)	Mortality (n, %)	AKI (n, %)	Neurolo-gical injury (n, %)
	MH/DH	MH/DH	MH/DH	MH/DH	MH/DH	MH/DH	MH/DH	MH/DH	MH/DH	MH/DH	MH/DH	MH/DH	MH/DH
Abdul-Khaliq et al.	102 (41)/220 (90)	NR/NR	22–35/<18	0 (0)/0 (0)	29 (100)/18 (100)	0 (0)/0 (0)	NR/NR	NR/NR	NR/NR	NR/NR	NR/NR	NR/NR	NR/NR
Rastan et al.	156 (27.8)/208 (67.8)	89 (14.8)/110 (22.4)	25.3 (1.1)/19 (0.8)	0 (0)/0 (0)	47 (92.2)/40 (81.6)	4 (7.8)/9 (18.4)	8.4 (4.7)/12 (10.8)	89 (50)/126 (111)	132 (102)/146 (100)	31 (60.8)/47 (95.9)	0 (0)/1 (2)	0 (0)/2 (4.1)	2 (3.9)/6 (12.2)
Kornilov et al.	130 (41.5)/150 (46.7)	56 (20.7)/70 (34.8)	25 (1.5)/20 (0.7)	35 (100)/0 (0)	0 (0)/0 (0)	0 (0)/27 (100)	NR/NR	144 (194)/120 (106.7)	21.2 (19)/18.6 (20.2)	4 (11.4)/5 (18.5)	4 (11.4)/5 (18.5)	21 (60)/5 (18.5)	2 (5.7)/8 (29.6)
Xie et al.	146.9 (40.6)/189.6 (41.2)	75.9 (22.7)/88.9 (19.6)	25.7 (2.6)/18.8 (1.7)	61 (100)/0 (0)	0 (0)/0 (0)	0 (0)/53 (100)	4.6 (3)/9.2 (7.6)	43.6 (49.3)/93.3 (77.7)	63.8 (104.3)/69.2 (98.9)	24 (39.3)/23 (43.4)	6 (9.8)/4 (7.5)	2 (3.3)/1 (1.9)	3 (4.9)/10 (18.9)
Bottcher et al.	215.7 (65.8)/104 (122.1)	53 (20.4)/53 (19.8)	27.9 (0.2)/16 (3)	74 (100)/51 (100)	0 (0)/0 (0)	0 (0)/51 (100)	12 (9.8)/8.7 (6.9)	219.7 (165.6)/174.3 (162.5)	NR/NR	39 (52.7)/29 (56.9)	6 (8.1)/9 (17.6)	39 (52.7)/29 (56.9)	NR/NR
Percent-age (categor-ical values) or mean ± SD (contin-uous variables)	161.5 (47.8)/169.5 (80.4)	68.1 (20)/81.6 (23.3)	26.2 (1.6)/18.2 (1.9)	170 (68)/51 (25.8)	76 (30.4)/58 (29.3)	4 (1.6)/140 (70.7)	8.6 (6.9)/9.9 (8.6)	128.9 (127.9)/129.2 (119.7)	77.3 (90.7)/87.8 (89.1)	98 (44.3)/104 (57.8)	16 (7.2)/19 (10.6)	62 (28.1)/37 (20.6)	7 (4.8)/24 (18.6)

Abbreviations: NR = Not Reported, SD = Standard Deviation, MH = Moderate hypothermia, DH = Deep hypothermia, SD = Standard deviation, NR = Not reported, CPB = Cardiopulmonary bypass, SACP = Selective antegrade cerebral perfusion, FFCPB = Full-flow cardiopulmonary bypass, CA = Circulatory arrest, LOS = Length of stay, AKI = Acute kidney injury.

**Table 4.** Pooled estimates with SMDs for Continuous Variables and Odds Ratios for Categorical Variables.

​	Parameter	SMD or odds ratio (95% CI)	*p*
Continuous	Age	0.15 (−0.21–0.51)	0.41
	Weight	0.12 (−0.2–0.43)	0.46
	Minimum rectal temperature	4.92 (3.2–6.64)	<0.01
	CPB Time	−0.61 (−1.62–0.41)	0.24
	Cross-clamp Time	−0.55 (01.01–−0.08)	0.02
	ICU Stay	−0.29 (−0.99–0.42)	0.42
	Ventilation Time	−0.2 (−0.7–0.3)	0.43
	Chest drainage	−0.04 (−0.28–0.19)	0.72
Categorical	Arch pathologies	0.41 (0.12–1.39)	0.15
	VSD	0.99 (0.61–1.59)	0.97
	TGA	0.44 (0.03–5.75)	0.53
	DORV	0.64 (0.2–2.01)	0.44
	Use of FFCPB	1 (0.17–5.95)	0.2
	Use of SACP	35.3 (0.48–2586.4)	0.1
	Use of circulatory arrest	0 (0–0.34)	0.01
	Post-op complications	0.49 (0.19–1.21)	0.12
	Post-op AKI	1.49 (0.37–6.1)	0.58
	Post-op neurological injury	0.21 (0.09–0.51)	<0.001
	Early death (<30 days)	0.62 (0.3–1.26)	0.18

Abbreviations: CI = Confidence Interval, CPB = Cardiopulmonary Bypass, SMD = Standardized Mean Difference, VSD = Ventricular septal defect, TGA = Transposition of the great arteries, DORV = Double outlet right ventricle, FFCPB = Full-flow cardiopulmonary bypass, SACP = Selective antegrade cerebral perfusion, AKI = Acute kidney injury.

**Figure 1.**
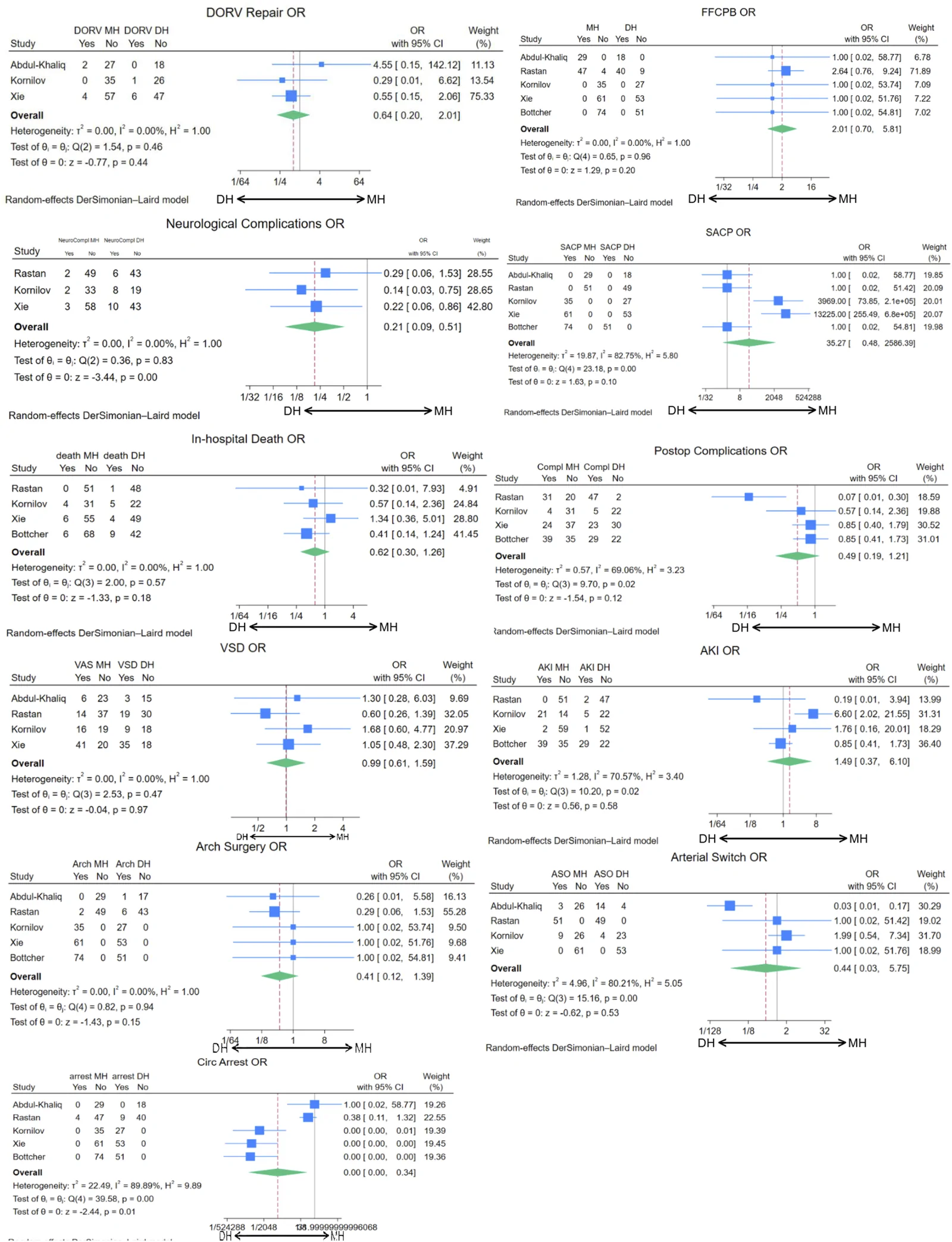
Meta-analyses with odds ratios for categorical variables and outcomes for MH vs. DH. OR = Odds ratio, DORV = Double outlet right ventricle, FFCPB = Full-flow cardiopulmonary bypass, SACP = Selective antegrade cerebral perfusion, VSD = Ventricular septal defect, AKI = Acute kidney injury.

**Figure 2.**
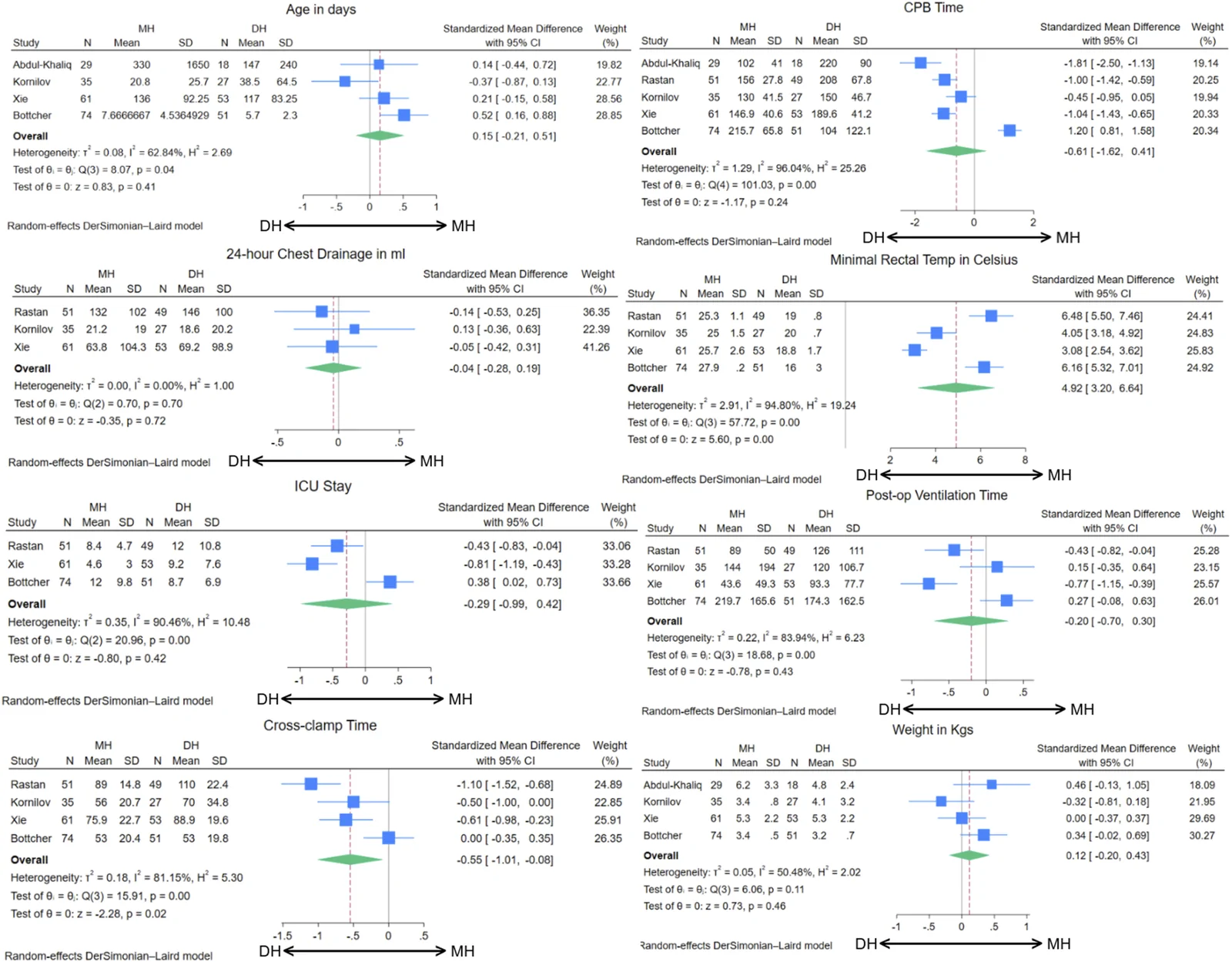
Meta-analyses with standardized mean differences for continuous variables and outcomes for MH vs. DH. CPB = Cardiopulmonary bypass, ICU = Intensive care unit.

### Risk of bias assessment

We used the ROBINS-I tool for the included non-randomized comparative studies. Overall risk of bias was deemed as moderate. A traffic light diagram and summary are provided in Supplemental Figure 2. Publication Bias was assessed using the Egger’s test and funnel plots. No evidence of publication bias was found, as Egger’s test *p*-values were >0.05 in both neurological outcomes and X-Clamp time (Supplemental Figure 3).

### Demographics and medical history

Given the available information from the included studies, data for the following demographic and medical history features for MH vs. DH were extracted: age (96.3 vs. 68.3 days, *p* = 0.41), body weight at time of surgery (4.4 vs. 4.3 kg, *p* = 0.46), preoperative diagnosis of aortic arch hypoplasia and coarctation (77.8% vs. 76.7%, *p* = 0.15), ventricular septal defect (34.8% vs. 36.7%, *p* = 0.97), transposition of the great arteries (28.5% vs. 37.2%, *p* = 0.53), and double outlet right ventricle (2.7% vs. 3.9%, *p* = 0.44) (Tables 1, 2, and 4).

### Perioperative details and outcomes

Similarly, for the peri-operative stage, we extracted data for MH vs. DH about the average intraoperative rectal temperature (26.2°C vs. 18.2°C, *p* < 0.001), cardiopulmonary bypass time (161.5 vs. 169.5 min, *p* = 0.24), cross-clamp time (68.1 vs. 81.6 min, *p* = 0.02) use of SACP (68% vs. 25.8%, *p* = 0.1), use of full-flow CPB (30.4% vs. 29.3%, *p* = 0.2) and the use of circulatory arrest (1.6% vs. 70.7%, *p* = 0.01).

In terms of early postoperative outcomes, we studied the overall rate for in-hospital mortality (7.2% vs. 10.6%, *p* = 0.18), in-hospital (30-days) complications (44.3% vs. 57.8%, *p* = 0.12), post-operative incidence of acute kidney injury (28.1% vs. 20.6%, *p* = 0.58), length of stay in the ICU (8.6 vs. 9.9 days, *p* = 0.42), postoperative ventilation time (128.9 vs. 129.2 h, *p* = 0.43), chest drainage volume in the first 24 h after surgery (77.3 vs. 87.8 ml, *p* = 0.72) and 30-days neurological complication rates (4.8% vs. 18.6%, *p* < 0.001). The final term resulted from pooling the outcome rates for any neurological event, like cerebrovascular accidents (stroke, TIA), seizures, delayed recovery of consciousness, coma, changes in MRI or CT of the brain, brain edema, and cerebral hemorrhage. Most studies reported on neurological impairment events in cumulative terms like this. The use of circulatory arrest and the incidence of cumulative neurological complications were increased in the DH group.

## Discussion

This meta-analytic synthesis of 448 patients represents a comprehensive evaluation of hypothermia techniques in pediatric cardiac surgery. Compared to a previous analysis by Alkhatip et al.^1^ which assessed the outcomes after DHCA with EEG monitoring in a single-arm manner, our work aimed to directly compare deep to moderate hypothermia in pediatric cardiac surgery patients. We found that preoperative details and demographic characteristics were comparable between groups. Circulatory arrest was used more frequently and cross-clamp times were significantly longer in the DH cohort; however, 30-days postoperative complication rates, mortality, and acute kidney injury were similar between groups. Notably, our data suggest that postoperative neurological impairment may be more prominent in the DH group.

In our analysis of perioperative morbidity, for the majority of perioperative outcomes no significant differences were observed between patients managed with deep versus moderate hypothermia. These findings were consistent across comparable surgical procedures and diagnoses, with similar CPB durations and operative characteristics, consistent with our findings, other studies have reported comparable perioperative outcomes in pediatric patients managed with DH versus MH.^1,3^ Rastan et al. demonstrated that MH was associated with shorter intensive care unit stays, reduced need for inotropic support, and improved pulmonary recovery without compromising overall outcomes.^19^ Other studies have shown DH to be associated with hypothermia-induced coagulopathy, cerebral microvasculature endothelial dysfunction, neuronal damage, increased systemic inflammatory response, and increased risk of organ dysfunction.^22,23,24,25,26^ There is evidence that MH provides inadequate protection to the lower half of the body, leading to reduced oxygen delivery and subsequent renal insufficiency.^27,28^

In terms of the ideal cerebral perfusion and protection strategy, no study has identified a single method that clearly surpasses the results of all the others.^29^ Starr et al. revealed a significant shift from hypothermic circulatory arrest to cerebral perfusion with an increase in mean nadir temperature from 23.9°C to 25.6°C.^10,30^ This tendency to avoid DHCA has led to the introduction and widespread utilization of new support techniques such as regional cerebral perfusion despite a lack of data showing superiority of regional cerebral perfusion to DHCA.^31,32,33^ A study looking at MH + SACP suggests that SACP attenuates the metabolic changes seen after circulatory arrest, which was deemed to contribute to the development of brain injury during aortic surgery.^34^ First described by Asou in 1996,^35^ SACP is preferred in many adult and pediatric centers as it provides continuous blood supply to the brain throughout surgery, which may allow one to tolerate increased metabolic rates at higher body temperatures in the range of 25–26°C. The duration of cooling and rewarming are shortened, and consequently, the CPB duration and CPB-associated adverse events may be reduced.^36,37,38^ On the other hand, not without its drawbacks, SACP has been associated with focal infarcts in central regions of the brain^7,39^ and increased rates of postoperative renal dysfunction compared to DHCA.^7^

The prevalence of postoperative neurological injury after pediatric cardiac surgery is 2-11%.^40^ In the present study, this term pooled all postoperative neurological adverse events, like cerebrovascular accidents, transient ischemic attacks (TIA), evidence of cerebral injury on MRI imaging, intracerebral hemorrhage, and seizures, and the overall rate was 4.8% for MH and 18.6% for DH (*p* < 0.001). Interestingly, several other studies, including an RCT, failed to demonstrate a significant difference in early neurological outcomes between DH and MH.^41,42^ Starr et al. also showed comparative neurological injury rates between the two techniques when looking only at pediatric patients undergoing arch surgery.^10^ A possible explanation for the possible superiority of MH over DH in terms of early neurological impairment rates found in our study may be due to the broad, heterogenous, and early orientation of the neurological outcomes used in the included trials. All of these outcomes are significant to patients, parents and caregivers, but their contribution toward long-term neurodevelopmental outcomes is uncertain. Fuller et al. performed a planned analysis of infants enrolled in a prospective study looking at the effect of DHCA on a neurodevelopmental assessment at 4 years.^43^ In that cohort of children undergoing repair of heart disease in infancy, the use of DHCA for repair of single-ventricle and biventricular heart disease without aortic arch obstruction was not predictive of worse performance for any neurodevelopmental domain. It may not be the temperature or the circulatory arrest in itself that leads to worse neurodevelopmental outcomes. As has been known, for some time, there is a safe duration of deep hypothermia and circulatory arrest. Compared to a previous analysis by Alkhatip et al.^1^ which assessed the outcomes after DHCA with EEG monitoring in a single-arm manner, our work aimed to directly compare deep to moderate hypothermia in pediatric cardiac surgery patients. A longer duration of DHCA was found to lead to more EEG seizures, which were associated with increased neurologic abnormalities and less optimal neurodevelopmental outcomes.^1,44^ Finally, although the difference in age at surgery between groups did not reach statistical significance, the DH cohort was numerically younger (mean 68.3 vs. 96.3 days), and younger age at the time of cardiac surgery is a well-established independent risk factor for postoperative neurological injury. This age disparity, while not statistically significant in our limited sample, may have contributed to the higher neurological complication rate observed in the DH group and represents an important potential confounder.

The observed heterogeneity of published results proves the multifactorial etiology of clinical outcomes after pediatric cardiac surgery with either MH or DH, besides the level of the minimum intraoperative temperature itself. A good instance is the variability of cerebral blood flow velocity during CPB, which is dependent on age and weight.^20^ In addition, contrary to the previous belief that postoperative neurological injury is only linked to intraoperative factors, like CPB duration, use of DHCA, cooling, blood gas management, and hemodilution rates,^39,45,46^ fixed variables like sex, race, socioeconomic status, and birth weight are of significant relevance as well.^47^ Outcomes also vary significantly across centers, suggesting that institutional experience, surgical volume, and subtle differences in perfusion strategies may contribute to the observed heterogeneity.^10,48^ Overall, the wide variability in reported outcomes highlights the need for more standardized reporting and multicenter analyses to better delineate the relative contributions of patient characteristics and institutional practices to early postoperative outcomes.

A significant consideration affecting the interpretation of our primary neurological findings is the substantial heterogeneity in how neurological outcomes were defined and reported across the included studies. While some studies reported neurological events as a single composite endpoint without further specification, others provided detailed classifications including seizures, intracranial hemorrhage, delayed recovery of consciousness, and imaging-confirmed stroke. This variability mirrors broader challenges in the field, as no standardized definition for neurological injury after pediatric cardiac surgery currently exists. Pooling heterogeneous endpoints such as seizures, delayed awakening, MRI abnormalities, and stroke into a single composite may overestimate the clinical significance of the observed difference, as transient or subclinical findings carry different prognostic implications than permanent deficits. In addition, another critical interpretive consideration is that the comparison between DH and MH in the included studies is confounded by concurrent differences in perfusion strategy. Approximately 71% of DH patients underwent circulatory arrest, compared with only 1.6% of MH patients, while all MH patients received some form of cerebral perfusion. Therefore, the observed neurological differences may reflect the effects of circulatory arrest, the protective effects of continuous cerebral perfusion, the temperature differential, or a combination of these factors. Furthermore, neurodevelopmental impairment, which is the outcome of greatest concern to families and clinicians, often manifests months to years after surgery, and early postoperative neurological events do not reliably predict long-term neurodevelopmental trajectories. Notably, Fuller et al. found that DHCA was not predictive of worse neurodevelopmental performance at 4 years, suggesting that early neurological differences may not translate into lasting functional deficits.^43^

Finally, several additional limitations warrant consideration when interpreting these findings. First, the applicability of our results is constrained by the absence of age-stratified analyses; the included studies pooled neonates, infants, and older children despite known differences in cerebral vulnerability, metabolic demands, and surgical complexity across these developmental stages. Second, as discussed above, the lack of a uniform definition for neurological injury across studies introduces measurement heterogeneity that may have influenced the pooled effect estimates. Third, the included studies did not provide sufficient granularity regarding the duration of DHCA, precluding stratified analyses by DHCA time; a variable known to significantly impact neurological outcomes, with durations exceeding 40 min associated with adverse neurodevelopmental outcomes.^49^ Fourth, the relatively small total sample size of 448 patients limits statistical power, particularly for detecting differences in less common outcomes such as mortality. Fifth, the observational and retrospective nature of all included studies, most of which lacked propensity-score matching or other rigorous methods to control for confounding, introduces potential selection bias, as study-level data do not permit individual patient-level adjustment for potential confounding factors. Finally, the absence of long-term neurodevelopmental follow-up data prevents assessment of whether the observed early neurological differences translate into meaningful functional impairments at school age or beyond, a critical consideration given that early brain MRI abnormalities do not consistently predict neurodevelopmental outcomes before 2 years of age.^49,50^

## Conclusion

This meta-analysis evaluated early postoperative outcomes in pediatric patients undergoing cardiac surgery with either moderate or deep hypothermia. Moderate hypothermia with regional perfusion may be associated with a lower incidence of early postoperative neurological events compared to deep hypothermia, while overall in-hospital mortality and morbidity appear comparable between strategies. These findings support moderate hypothermia with regional perfusion as a clinically feasible and well-tolerated alternative to deep hypothermia in pediatric cardiac surgery. However, these findings are limited by the small number of included studies, the observational study designs, the heterogeneity of neurological outcome definitions, and the influence of confounding factors. They also pertain exclusively to early postoperative outcomes; whether the observed neurological differences translate into long-term neurodevelopmental impairment remains unknown and warrants dedicated follow-up studies.

## Supplemental material


Supplemental material - Deep versus moderate hypothermia in pediatric cardiac surgery: Systematic review and meta-analysis of early postoperative outcomes
Supplemental material for Deep versus moderate hypothermia in pediatric cardiac surgery: Systematic review and meta-analysis of early postoperative outcomes by Ioannis Zoupas, Jared M. Covell, Julieta Cruz Naranjo, Blaz Podgorsek, Peter C. Chen, Damien J. LaPar and Christopher E. Greenleaf in Perfusion

## Data Availability

The data underlying this study were obtained from publicly accessible sources. These data are available from the respective data repositories subject to their standard application and data use agreements.*
